# Supplementary material for: The AMPK-related kinase NUAK1 controls cortical axons branching by locally modulating mitochondrial metabolic functions
Source: Nat Commun. 2024 Mar 21;15:2487. doi: 10.1038/s41467-024-46146-6 (PMC10958033; doi:10.1038/s41467-024-46146-6)
Supplement: Supplementary file 1 — Supplementary Information [file 41467_2024_46146_MOESM1_ESM.pdf]

The AMPK-related kinase NUAK1 controls cortical axons branching by locally modulating mitochondrial metabolic functions

Marine Lanfranchi, Sozerko Yandiev, Géraldine Meyer-Dilhet, Salma Ellouze, Martijn Kerkhofs, Raphael Dos Reis, Audrey Garcia, Camille Blondet, Alizée Amar, Anita Kneppers, Hélène Polveche, Damien Plassard, Marc Foretz, Benoit Viollet, Kei Sakamoto, Rémi Mounier, Cyril F Bourgeois, Olivier Raineteau, Evelyne Goillot and Julien Courchet

Supplementary material

SUPPLEMENTARY FIGURES

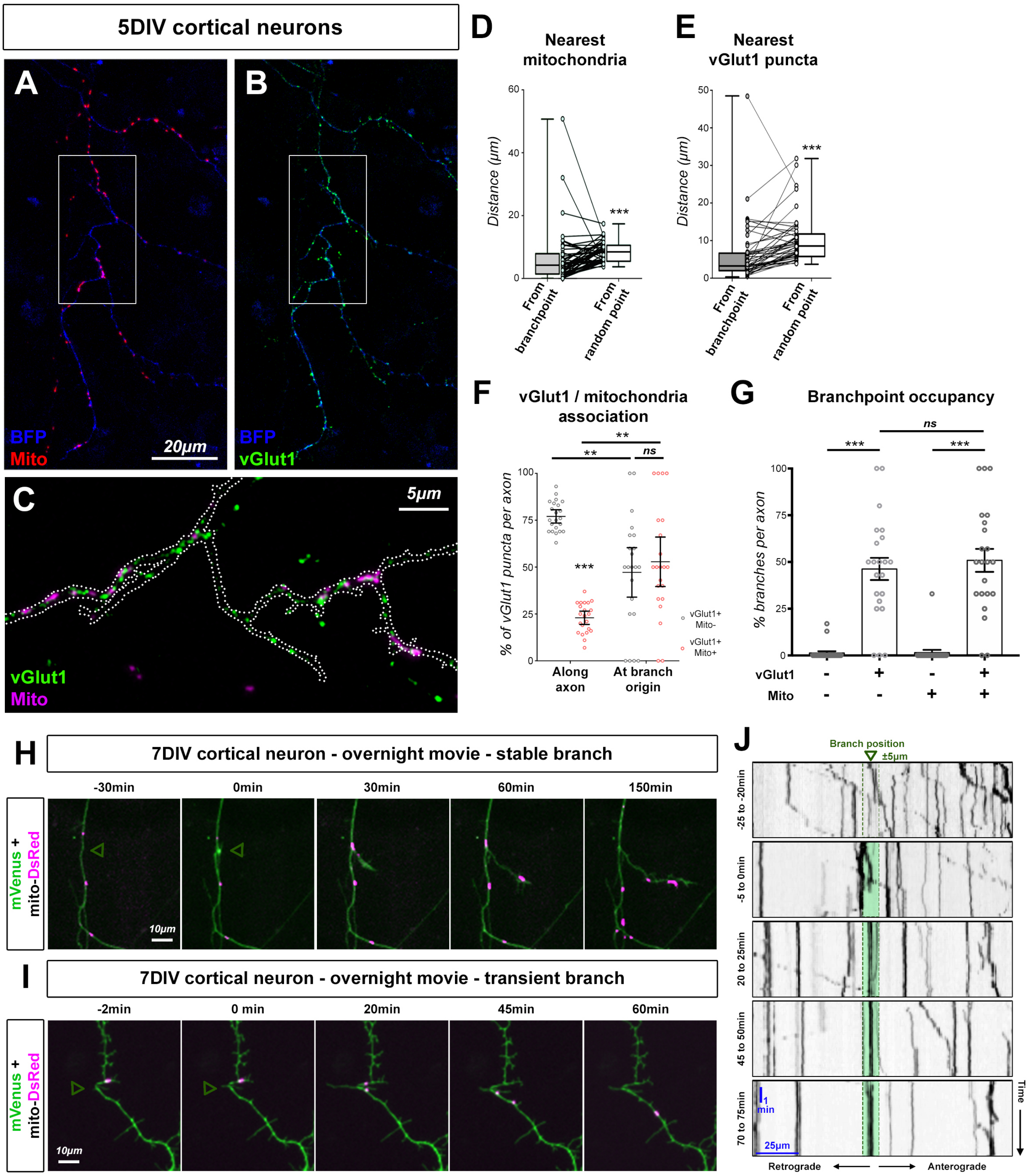

Supplementary Figure 1 (related to Figure 1): dynamic correlation between presynaptic vesicle and mitochondria positioning and branch formation

(A-B) Representative images of a 5DIV cortical neuron following expression of the mitochondrial marker mito-DsRed (A) or the presynaptic marker vGlut1-GFP (B). BFP was used to visualize neuronal morphology. Magnification of a segment of axon (C) shows vGlut1-GFP and mito-DsRed localization respective to branch origin (white dotted line).

(D-E) Observed minimal distance from a branchpoint compared to average distance from a random point along the axon, relative to the nearest mitochondrion (D) or synaptic puncta (E). Box-plot: 75th percentile, median and

25th percentile. Individual dots represent the average for one axon. Statistical tests: Wilcoxon matched-pairs ranked test. $N_{(mito)}$=46, $N_{(vGlut1)}$=48 out of 3 independent neuronal cultures.

(F) Proportion of vGlut1 puncta devoid (grey) or associated to (red) mitochondria along the axon, and at branchpoints. Data: median ± 95% CI. Statistical tests: two-way ANOVA with Bonferroni's multiple comparison test. N=22 axons out of 2 independent experiments.

(G) Quantification of branchpoint occupancy by either vGlut1+ puncta, mitochondria, or vGlut1+ puncta and mitochondria. Each point represents the value (%) for a given axon. Data: average ± SEM. Statistical tests: Repeated measures ANOVA with Dunnett's multiple comparison test. N=22 axons out of 2 independent experiments.

(H-I) Examples of axons at 7DIV showing the distribution of mitochondria (magenta) before and after branch formation. Future branch position is indicated by a green arrowhead. Note the absence of mitochondria at the site of branch emergence prior to branch formation. mVenus was used as a space filler. (H) Stable branch typically recruited mitochondria at their origin and rapidly had mitochondria entering the branch. Eventually in some instances we could observe the pool of mitochondria at the branch origin moving into the branch. (I) On the contrary transient (unstable) branches failed to recruit and stabilize mitochondria at their origin.

(J) Kymographs of axonal mitochondria centered around branch position (green arrowhead) from axon in (Fig. 1C).

ns: not significant, *: P ≤ 0.05, **: P ≤ 0.01, ***: P ≤ 0.001. Source data are provided as a Source Data file.

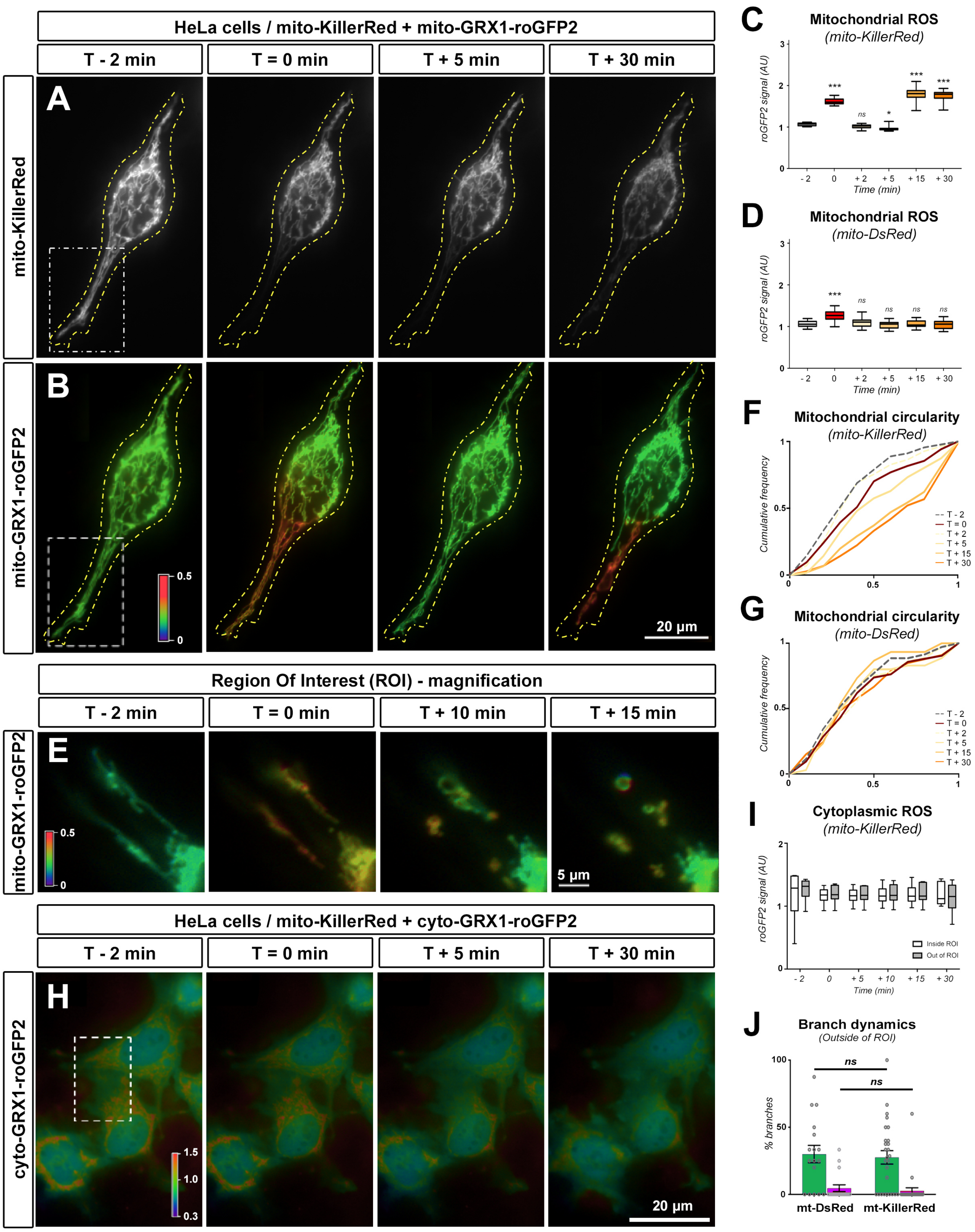

Supplementary Figure 2 (related to figure 1): spatial and temporal validation of mitochondria photoinactivation using KillerRed

(A-B) Example of a HeLa cell expressing mt-KR (A) and mito-GRX1-roGFP2 (B) before and after photoinactivation of mitochondria in the ROI (white box). Laser intensity 100%, 30 seconds. Cell boundary

indicated in yellow. mito-GRX1-roGFP2 is displayed as a fluorescence ratio between 405 and 488 illumination using a Rainbow LUT (Red: high ratio, oxidized. Blue: low ratio, reduced).

(C-D) Quantification of mitochondrial ROS accumulation (roGFP2 fluorescence ratio 405:488, normalized to T:-3 min, higher means more oxidized) at given time-points before, during and after CALI. Laser intensity 100%, 30 seconds. HeLa cells were co-transfected with mito-GRX1-roGFP2 and either mt-KR or the control mito-DsRed, as indicated. Box-plot: 75th percentile, median and 25th percentile. Analysis: Repeated measures ANOVA with Dunnett's multiple comparison test. $N_{mt\text{-}KR}$=11 cells, $N_{mt\text{-}DsRed}$=29 cells.

(E) Magnification of the ROI of a HeLa cell before and after CALI showing mitochondria fragmentation and rounding.

(F-G) Mitochondria circularity was measured in the ROI before and after CALI in HeLa cells. Graphs show the cumulative circularity of mitochondria in cells expressing mt-KR (F) or mt-DsRed (G). $N_{mt\text{-}KR}$=9 cells. $N_{mt\text{-}DsRed}$=9 cells. Values of individual mitochondria $N_{mt\text{-}KR}$ 90 to 150 individual mitochondria. $N_{mt\text{-}DsRed}$ 30 to 40 individual mitochondria

(H) Representative image of HeLa cells co-transfected with mt-KR and cyto-GRX1-roGFP2. roGFP2 signal is displayed as a fluorescence ratio between 405 and 488 illumination using a Rainbow LUT (Red: high ratio, oxidized. Blue: low ratio, reduced). CALI was performed in the indicated ROI (white box). Laser intensity 100%, 30 seconds.

(I) Quantification of cytoplasmic ROS accumulation by measuring cyto-GRX1-roGFP2 signal inside and out of the ROI showed no significant leakage of ROS in the cytoplasm of cells following photostimulation of mitochondria-targeted KillerRed. Box-plot: 75th percentile, median and 25th percentile. $N_{mt\text{-}KR}$=7 cells. $N_{mt\text{-}DsRed}$=7 cells.

(J) Comparison of the percentage of growing/new branches (green) and retracting branches (magenta) out of the ROI in neurons electroporated with mito-DsRed or mito-KillerRed. Branch dynamics was observed over 12 hours. Data: average ± SEM. Analysis: two-way ANOVA with Bonferroni's multiple comparison test. N_{DsRed}=17 axons, $N_{KillerRed}$=27 axons out of 4 independent experiments.

ns: not significant, *: P ≤ 0.05, **: P ≤ 0.01, ***: P ≤ 0.001. Source data are provided as a Source Data file.

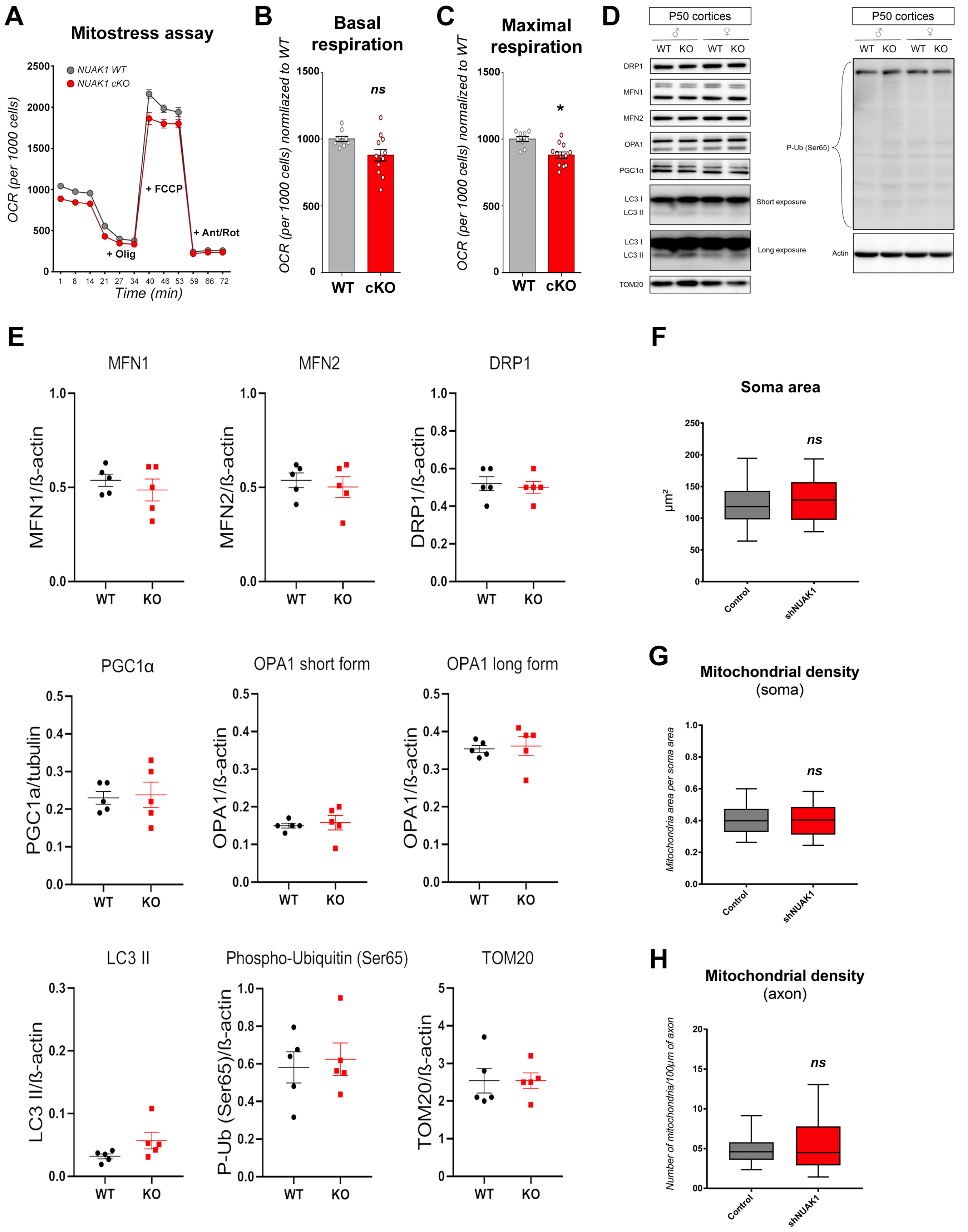

Supplementary Figure 3 (related to figure 3): mitochondrial phenotypes in cortices of NUAK1 cKO mice

(A) Measurement of oxygen consumption rate in 5DIV neuronal cultures from $Nex^{+/+}$;$NUAK1^{F/F}$ (WT) or $Nex^{CRE/+}$;$NUAK1^{F/F}$ (cKO) embryos. OCR values are normalised to cell number. Basal and maximal respiration values are represented in (B) and (C) respectively. N_{WT}=9, N_{cKO}=12 out of 4 independent experiments. Statistical test: Kruskal-Wallis test with Dunn's post-test. Data: average + SEM.

(D) Representative images of Western blots of protein extracts from Nex$^{+/+}$;NUAK1$^{F/F}$ (WT) or Nex$^{CRE/+}$;NUAK1$^{F/F}$ (cKO) mouse cortices at P50. (E) Quantifications of Western blots shown in D normalized to beta-actin or tubulin. Data: average + SEM. N_{WT}=5. N_{cKO}=5. Statistical test: Two-tailed Mann-Whitney. Original (uncropped) blots are provided in **Supplementary figure 10**.

(F-H) Quantifications of neuronal soma surface area (F), total surface area of somatic mitochondria (G), and density of axonal mitochondria (H) in WT and KO neurons. (F-G) $N_{Control}$=42, $N_{shNUAK1}$=37 out of 3 independent experiments. (H) $N_{Control}$=49, $N_{shNUAK1}$=57 out of 3 independent experiments. Statistical test: Two-tailed Mann-Whitney. Data: average + SEM.

ns: not significant, *: P ≤ 0.05, **: P ≤ 0.01, ***: P ≤ 0.001. Source data are provided as a Source Data file.

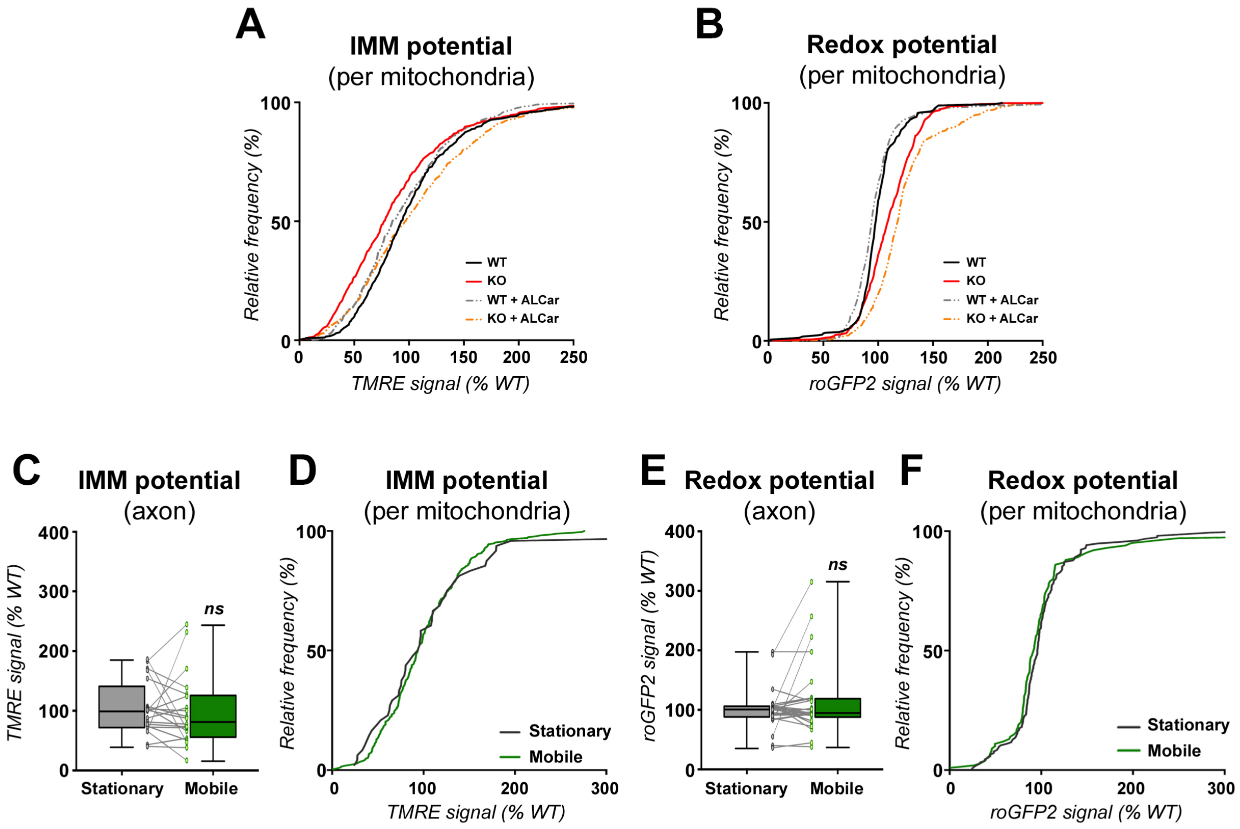

Supplementary Figure 4 (related to figure 3): mitochondrial membrane potential and redox potential are not correlated with mitochondrial motility

(A-B) Frequency distribution of individual mitochondria in the axon of NUAK1$^{F/F}$ neurons in conditions from Figure 5D-E. Plot of mitochondrial membrane potential (ΔΨm) (A) or redox potential (GRX1-roGFP2) (B).

(C-F) Effect of mitochondrial motility on IMM and redox potential in Wild-Type neurons. Time-lapse imaging was performed to sort out mobile (>5µm displacement) or stationary mitochondria. (C, E) Graphs represent the average values for axonal mitochondria in a given axon. Box-plot: 75th percentile, median and 25th percentile. Individual dots represent average (per axon). Statistical tests: Wilcoxon matched-pairs ranked test. $N_{(TMRE)}$=20, $N_{(roGFP2)}$=26 individual neurons of 5 independent neuronal cultures. (D, F) Frequency distribution of individual mitochondria in the axon.

ns: not significant, *: P ≤ 0.05, **: P ≤ 0.01, ***: P ≤ 0.001. Source data are provided as a Source Data file.

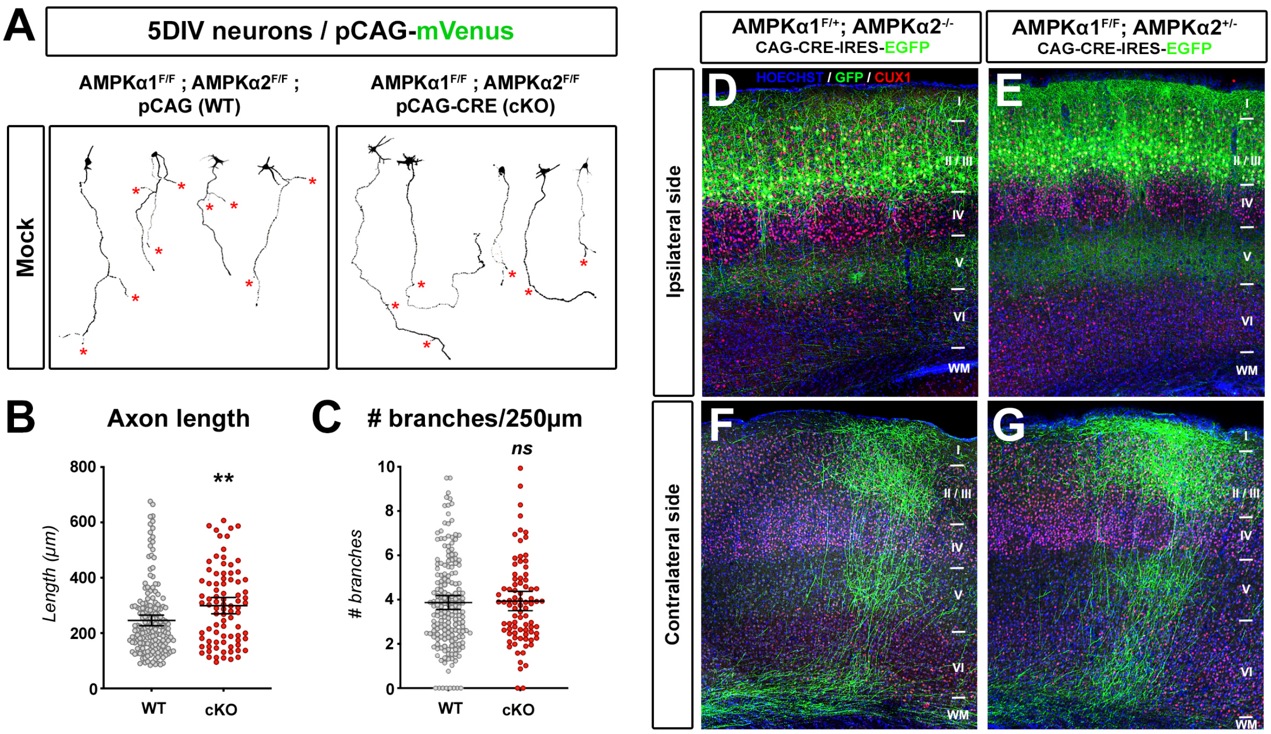

Supplementary Figure 5 (related to figure 4): reduction in AMPK activity does not impact cortical axon branching

(A) Representative images of cortical neurons from AMPKα1$^{F/F}$;AMPKα2$^{F/F}$ embryos and electroporated with either a control, empty vector (left) or a plasmid encoding CRE (right). Neuron morphology was achieved by expressing the green fluorescent protein mVenus. Red stars indicate collateral branches.

(B and C) Quantification of axonal length (B) and collateral branches (C) of neurons at DIV5 under the indicated conditions. Data: averages + 95 % CI. Statistical test: Mann-Whitney test. $N_{(WT)}$=174, $N_{(cKO)}$=85 individual neurons of 2 independent neuronal cultures.

(D-G) Representative images of coronal sections following *in utero* electroporation of CRE coding plasmid. Embryos were obtained from crosses between mice carrying a conditional allele for AMPKα1 (F/+) and a constitutive allele for AMPKα2 (+/-). These crosses enabled us to obtain animals in which the expression of AMPK was greatly reduced (AMPKα1$^{F/F}$; AMPKα2$^{+/-}$ or AMPKα1$^{F/+}$; AMPKα2$^{-/-}$). mVenus expression was used to visualize electroporated neurons. Immunohistochemistry with CUX1 (red) indicates superficial cortical layers. There was no effect of a reduction of AMPK activity on neurogenesis, neuron migration and ipsilateral axon branching (D; E) nor on terminal axon branching on the contralateral side (F; G).

ns: not significant, *: $P \leq 0.05$, **: $P \leq 0.01$, ***: $P \leq 0.001$. Source data are provided as a Source Data file.

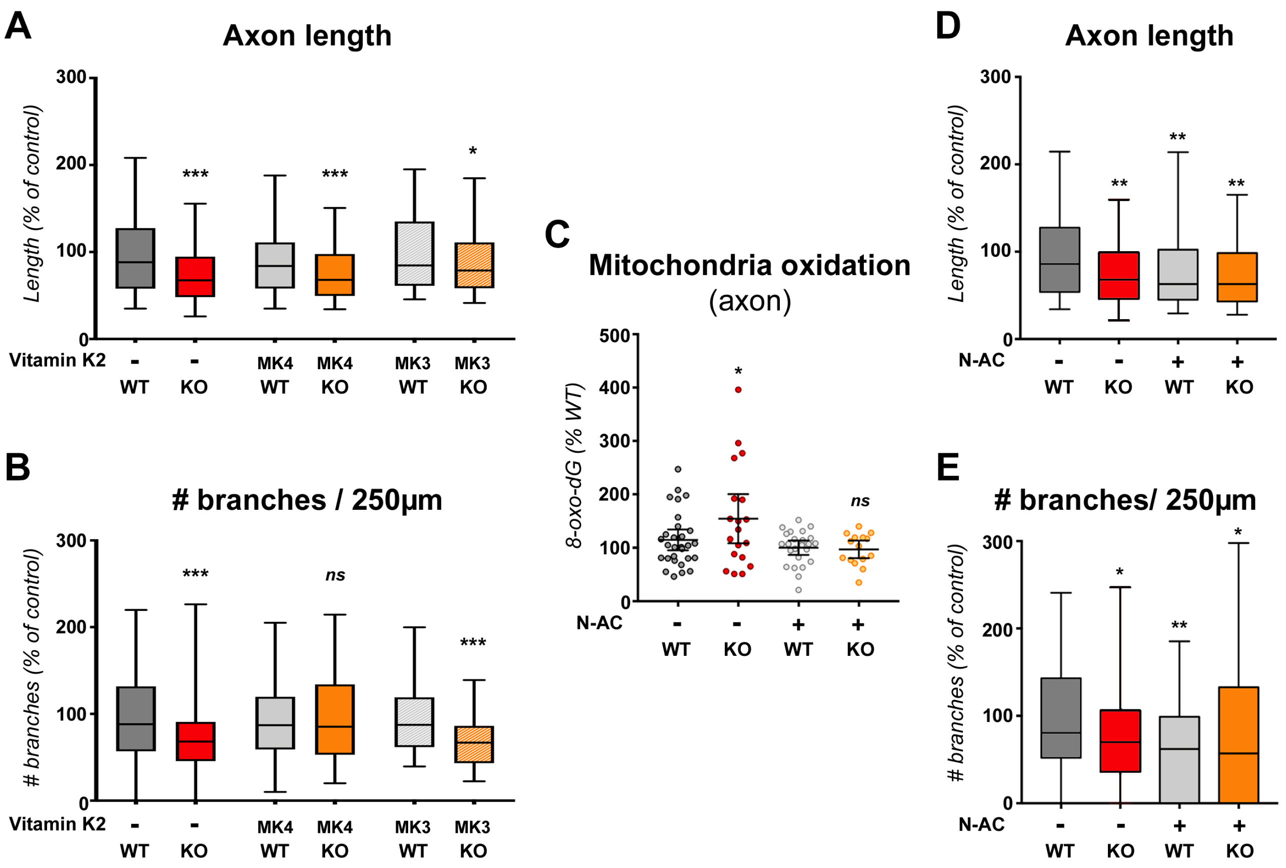

Supplementary Figure 6 (related to figure 5): differential effects of VK2 and N-AC on axon development

(A-B) Quantification of axon length and collateral branches of 5DIV neurons upon treatment with either Menaquinone-4 (MK4) (1µM), the active form of Vitamin K2, or the inactive for Menaquinone-3 (MK3) (1µM) from DIV2 to DIV5. Box-plot: 75th percentile, median and 25th percentile. Statistical tests: Kruskal-Wallis test with Dunn's post-test (each KO condition compared to the corresponding WT condition). $N_{(WT)}$=290, $N_{(KO)}$=164, $N_{(WT+MK4)}$=239, $N_{(KO+MK4)}$=147, $N_{(WT+MK3)}$=79, $N_{(KO+MK3)}$=76 out of 2 independent experiments.

(C) Measurement of mitochondrial DNA oxidation by immunostaining with 8-hydroxy-2-deoxyGuanosine (8-oxo-dG) antibody. Fixed neuronal cultures at 5DIV were stained with 8-oxo-dG and neuronal fluorescence was measured as a readout of oxidative stress. For each field imaged, electroporated neurons (GFP positive) signal was normalized to non-electroporated neurons to account for variations in signal to noise. Data: average ± 95% CI. Statistical tests: Kruskal-Wallis test with Dunn's post-test (each KO condition compared to the corresponding WT condition). $N_{(WT)}$=29, $N_{(KO)}$=19, $N_{(WT+N\text{-}AC)}$=24, $N_{(KO+N\text{-}AC)}$=15 out of 12 independent fields (2 independent cultures).

(D-E) Quantification of axon length and collateral branches of 5DIV neurons treated with N-AcetylCysteine (N-AC) (1mM) from DIV2 to DIV5. Box-plot: 75th percentile, median and 25th percentile. Statistical tests: Kruskal-Wallis test with Dunn's post-test (each condition compared to untreated WT condition). WT and KO conditions are the same as (A-B). $N_{(WT)}$=202, $N_{(KO)}$=83, $N_{(WT+N\text{-}AC)}$=168, $N_{(KO+N\text{-}AC)}$=68 out of 2 independent experiments.

ns: not significant, *: P ≤ 0.05, **: P ≤ 0.01, ***: P ≤ 0.001. Source data are provided as a Source Data file.

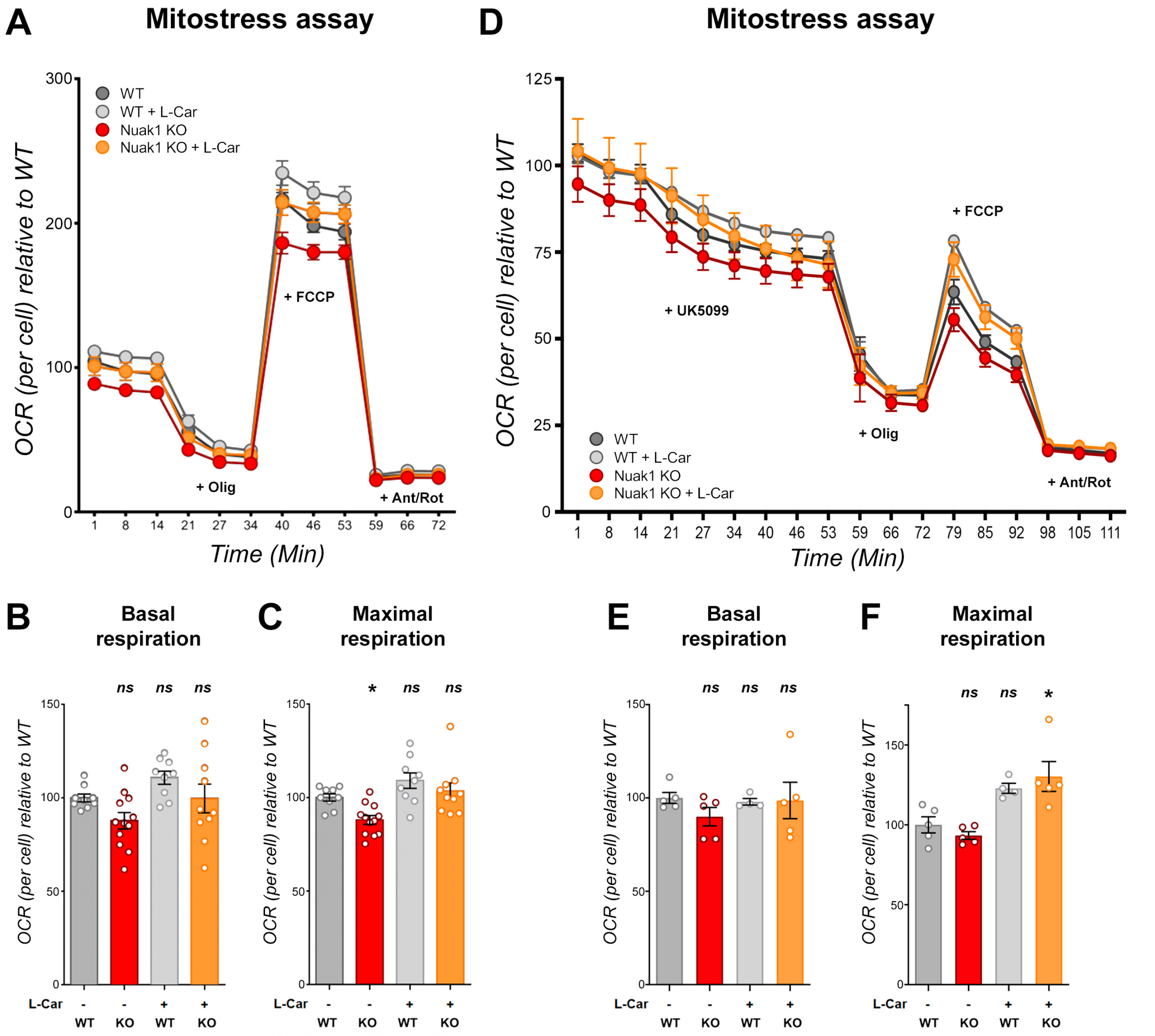

Supplementary Figure 7 (related to figure 5): L-Car treatment rescues mitochondrial respiration in NUAK1 cKO neurons

(A) Measurement of oxygen consumption rate in 5DIV WT or NUAK1 KO cortical neurons treated with L-Carnitine (1mM) (A). OCR values are normalised to cell number. Basal and maximal respiration values are represented in (B) and (C) respectively. N_{WT}=9, N_{KO}=12, $N_{WT+L\text{-}Car}$=9, $N_{KO+L\text{-}Car}$=10 out of 4 independent experiments. Statistical test: Kruskal-Wallis test with Dunn's post-test. Data: average + SEM.

(D) Measurement of oxygen consumption rate in 5DIV WT or NUAK1 KO cortical neurons treated with L-Carnitine (1mM) upon inhibition of pyruvate import inside mitochondria with UK5099. OCR values are normalized to cell number. Basal and maximal respiration values are represented in (E) and (F) respectively. N_{WT}=5, N_{KO}=5, $N_{WT+L\text{-}Car}$=4, $N_{KO+L\text{-}Car}$=5 out of 2 independent experiments. Statistical test: Kruskal-Wallis test with Dunn's post-test. Data: average + SEM.

ns: not significant, *: P ≤ 0.05, **: P ≤ 0.01, ***: P ≤ 0.001. Source data are provided as a Source Data file.

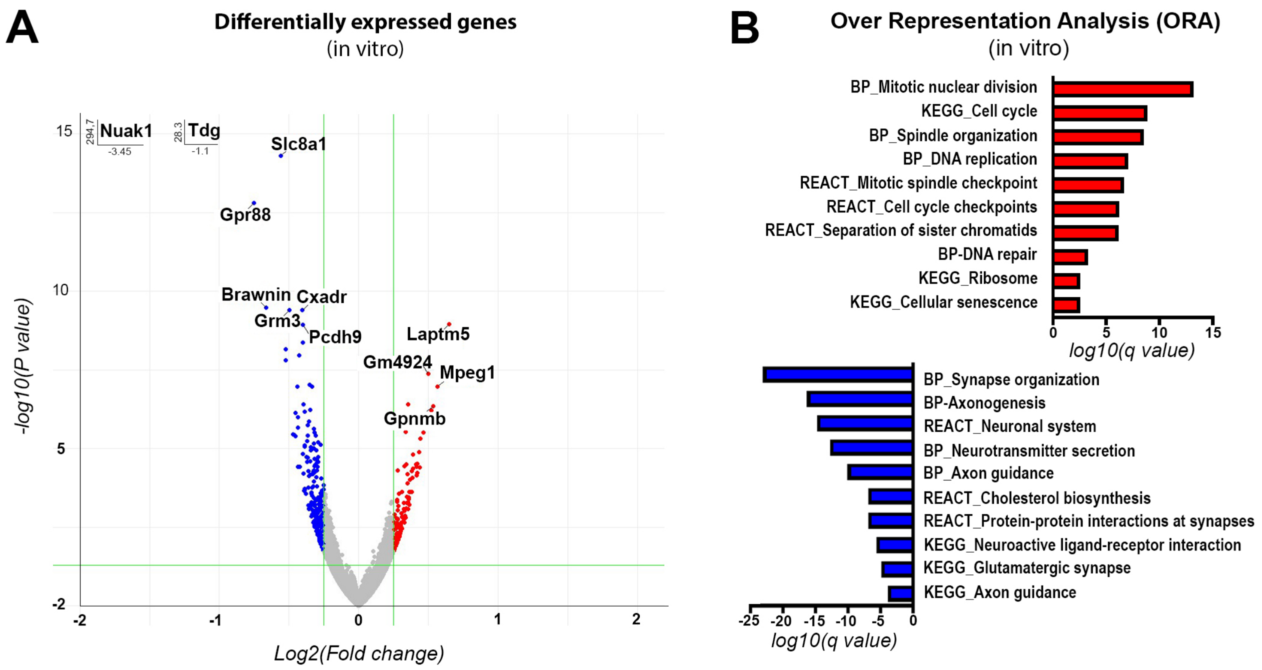

Supplementary Figure 8 (related to figure 6): Gene expression analysis of *Nuak1* knockout in cortical neurons

(A) Volcano plot showing the differentially expressed genes (DEG) between *Nuak1* KO and WT E15.5 cortical neurons following isolation and culturing 5 days in vitro. Significant DEGs (log2FC >< 0.25, p value < 0,05) appear in red (upregulated) or in blue (downregulated). Gene names appear for selected genes in NUAK1 KO vs WT mice. See table 1 for complete gene list.

(B) Bar plots representing selected over-represented GO BP, KEGG or Reactome categories, associated to transcripts upregulated (red) or downregulated (blue) in cultured *Nuak1* KO vs WT neurons.

See also **Supplementary data 1-2**

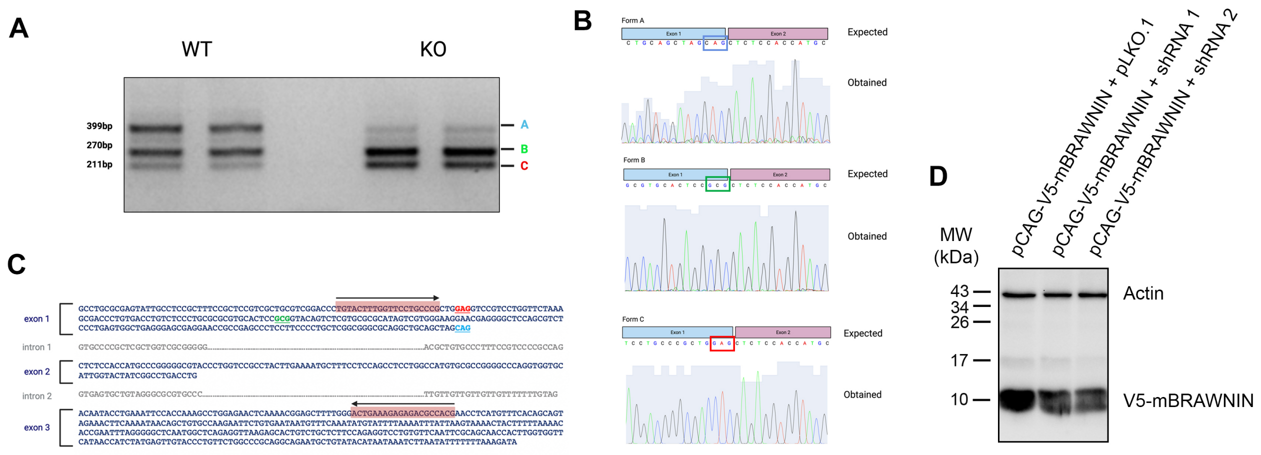

Supplementary Figure 9 (related to figure 6): Sequence confirmation of alternative splicing isoforms of mouse *Brawnin*

(A) Typical agarose gel following the amplification of *Brawnin* by PCR. PCR was done on total mRNA extracts samples following reverse transcription, from WT and NUAK1 KO 5DIV neurons. Each lane represents a separate culture. The three isoforms indicated with the predicted size were isolated and purified from the gel and sent for sequencing.

(B) Sequencing results for exon 1 – exon 2 junction sites for each *Brawnin* isoform isolated from an agarose gel. Sequences obtained were compared to and matched to the publicly available (Ensembl) sequences of *Brawnin*.

(C) Representation of exons (in blue) and introns (in grey) composing the mouse *Brawnin* DNA sequence. Three predicted alternative splicing donor sites are situated in the first exon and are highlighted in blue (form A), green (form B) and red (form C). These sites match with the exon-exon junction sites identified after sequencing. Primers used to amplify the fragments are highlighted in pink.

(D) Validation of shRNA plasmids targeting mouse *Brawnin*. V5-tagged mouse Brawnin was expressed in HEK293T cells together with a control vector (pLKO.1) or two distinct shRNAs targeting *Brawnin*. Western blot with the indicated antibodies validating shRNA potency at decreasing BRAWNIN expression.

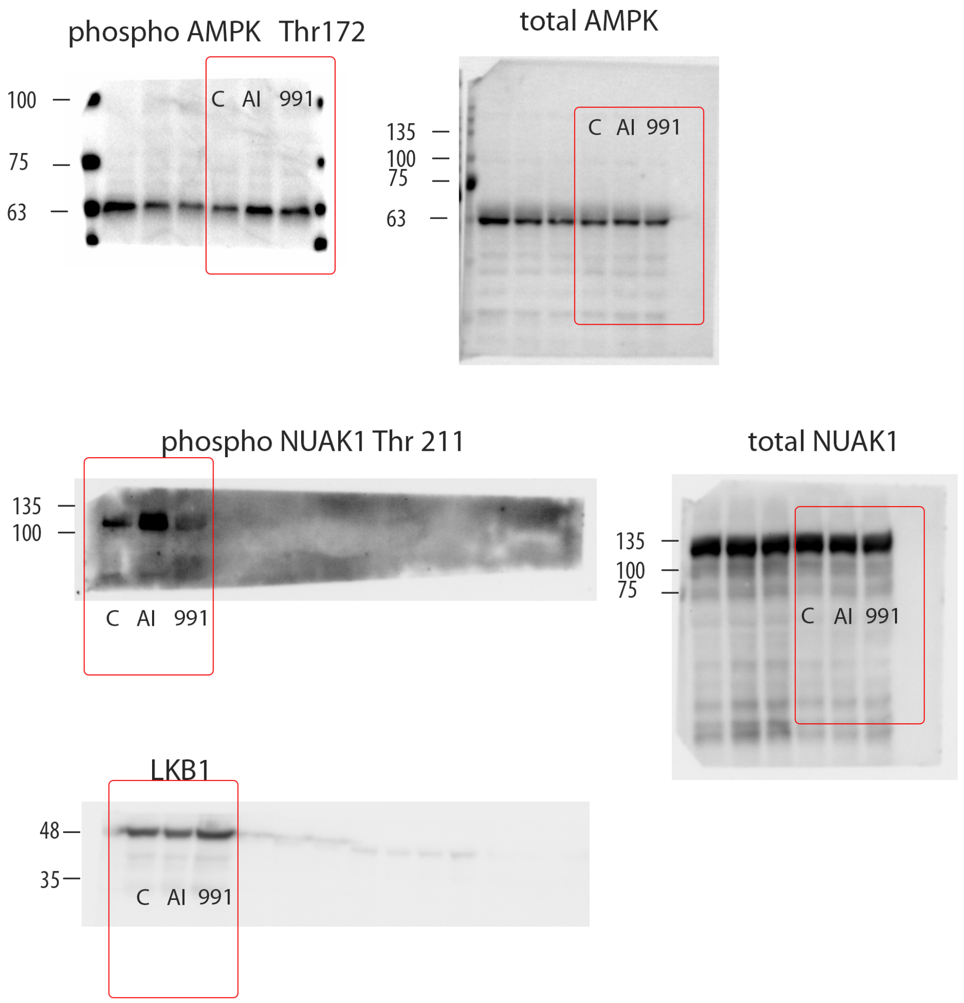

Supplementary Figure 10 (related to figure 4): uncropped Western blots used in this article (1/2)

Western-blots were imaged using the ChemiDoc Touch imager (Bio-Rad). Images were cropped and size was adjusted for making panel G in Figure 4. Contrast was not altered. Luminosity was adjusted for phospho NUAK1 Thr211 blot. C: control; AI: AICAR and 991 compound.

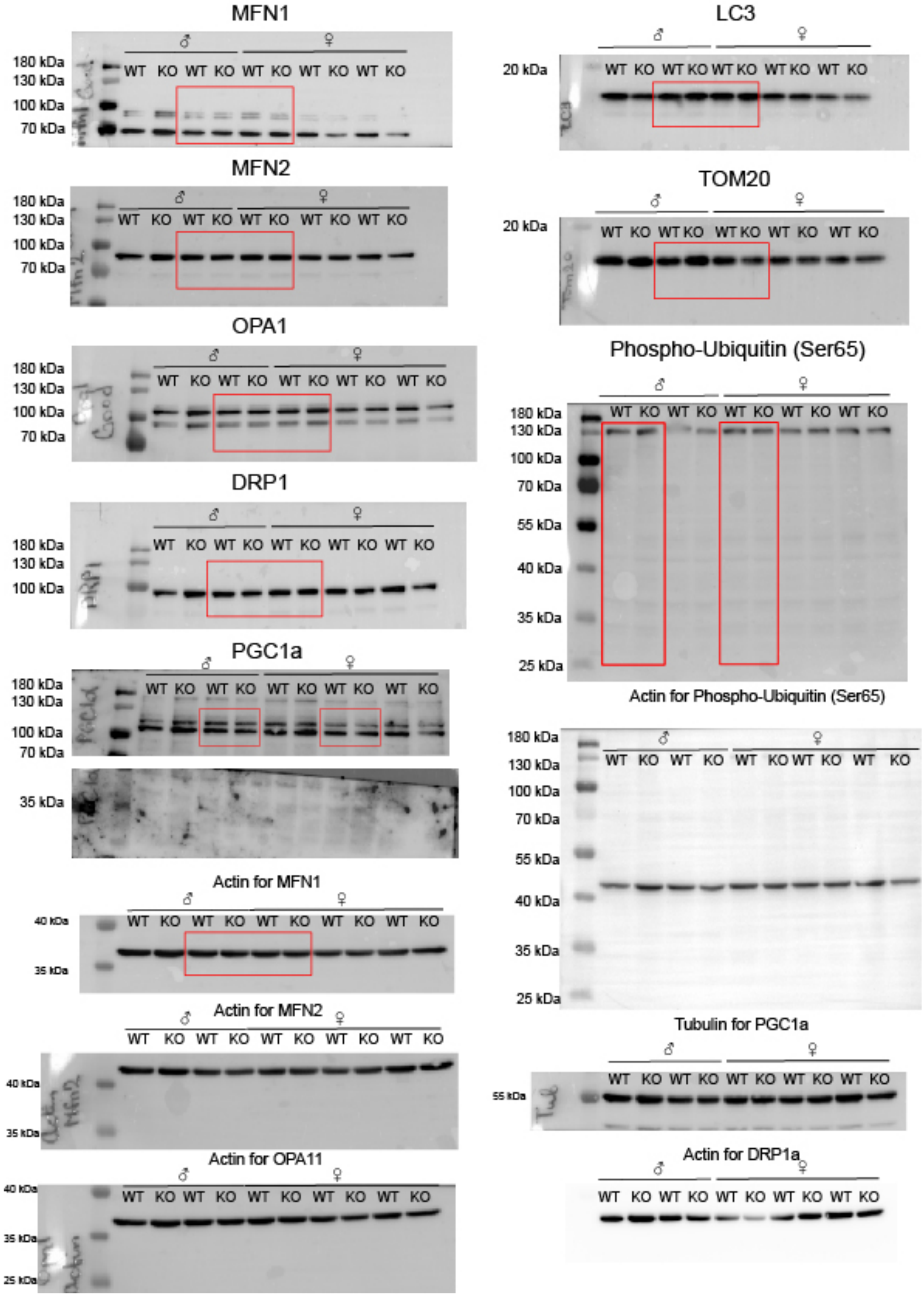

Supplementary Figure 10 (related to Supplementary Figure 3): uncropped Western blots used in this article (2/2)

Western-blots were imaged using the ChemiDoc Touch imager (Bio-Rad). Images were cropped and size was adjusted for making panel D in Supplementary Figure 3. Contrast and luminosity were not altered. WT and KO refer to Nex$^{+/+}$;NUAK1$^{F/F}$ and Nex$^{CRE/+}$;NUAK1$^{F/F}$ animals, respectively.

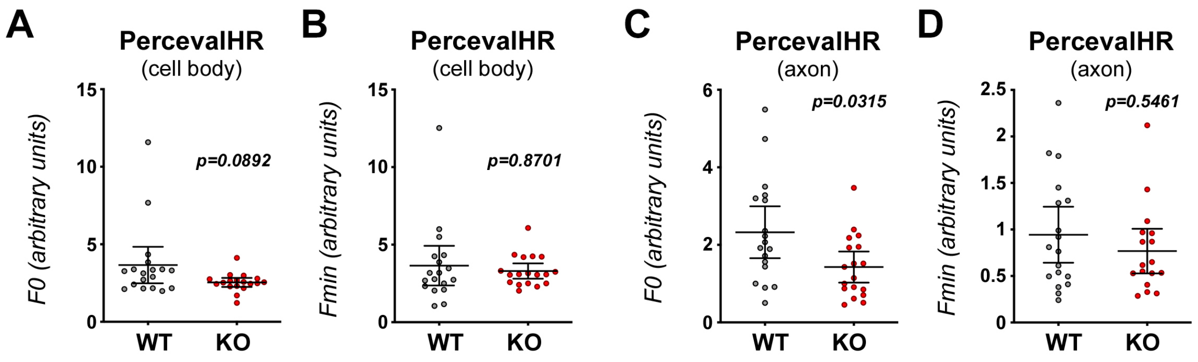

Supplementary Figure 11 (related to Figure 3): raw data of PercevalHR measurement in the soma and axon

Values (expressed as arbitrary units corresponding to the ratio $Perceval_{green}$ / $Perceval_{blue}$) before and after addition of FCCP in the soma (A-B) and in the axon (C-D). Data: average ± 95% CI. $N_{(WT)}$=17, $N_{(KO)}$=17 neurons out of 2 independent experiments. Statistical test: Two-tailed Mann-Whitney test.

ns: not significant, *: P ≤ 0.05, **: P ≤ 0.01, ***: P ≤ 0.001. Source data are provided as a Source Data file.

SUPPLEMENTARY METHODS

Animals

Mice breeding and handling was performed by following National Institutes of Health guidelines and the French and European legislation. Experimental protocols were approved by the CECCAPP Ethics committee (C2EA15) of the University of Lyon. Animals were used for breeding between 7 and 30 weeks of age. Animals were housed in standard conditions, kept in group and fed ad libitum with Safe A04 chow. Time-pregnant females were maintained in a 12hour light/dark cycle and obtained by overnight breeding with males of the same strain. Noon following breeding was considered as E0.5. Floxed *Nuak1* mice (*Nuak1*$^{tm1a(KOMP)Wtsi}$) have been described previously[1]. The NexCRE mouse line (*Neurod6*$^{tm1(cre)Kan}$)[2] were provided by Sandrine Humbert (Grenoble Institute of Neuroscience, France). The *Ampkα1*$^{-/-}$, *Ampkα2*$^{-/-}$ and *Ampkα*1$^{F/F}$; *Ampkα2*$^{F/F}$ mice have been described previously[3–5]. Animals were maintained on a C57Bl/6JRj background and animals for backcrosses were purchased from Janvier labs. Both sexes were used for cellular and molecular analysis without distinction except for RNA sequencing where sex was determined by PCR. For experiments including shRNA time-pregnant WT RjOrl:SWISS females were purchased from Janvier labs.

Number of animals per experiment:

1. Axonal length and branching analysis: At least 3 animals per condition.
2. Seahorse analysis: At least 4 animals per condition.
3. Analyses including fluorescent probes and biosensors: At least 3 animals per condition.
4. Western Blot analysis: 5 animals per condition.
5. In vivo axonal projection analysis: at least 5 animals per condition.
6. Colocalization analyses: At least 3 animals per condition.
7. RNA sequencing and related analysis: 4 animals per condition.

Genotyping:

The following primers were used for genotyping:

1) For CRE detection (CRE band: ~350 bp, unspecific band: ~250 bp)

CRE-F: 5’-GAACCTGATGGACATGTTCAGG-3’

CRE-R: 5’-AGTGCGTTCGAACGCTAGAGCCTGT-3’

MYO-F: 5’-TTACGTCCATCGTGGACAGC-3’

MYO-R: 5’-TGGGCTGGGTGTTAGCCTTA-3’

2) For NUAK1 conditional allele detection (WT band: 216 bp, Floxed band: 320 bp)
NUAK1F_5_F: 5'-GTCTGTTACGCGCTTTGTAATG-3'
NUAK1F_5_R: 5'-AGCATTTAGTTCCTTGTGAGGC-3'
3) For NUAK1 constitutive allele detection (LacZ band: 390bp, unspecific band: 200 bp)
LacZ_F: 5'-GTTGCAGTGCACGGCAGATACACTTGCTGA-3'
LacZ_R: 5'-GCCACTGGTGTGGGCCATAATTCAATTCGC-3'
Fabpi-200-F: 5'-TGGACAGGACTGGACCTCTGCTTTCCTAGA-3'
Fabpi-200-R: 5'-TAGAGCTTTGCCACATCACAGGTCATTCAG-3'
4) For AMPKα1 conditional allele detection (Floxed band: 310bp, WT band: 260 bp)
AMPKA1_Flox_F: 5'-TATTGCTGCCATTAGGCTAC-3'
AMPKA1_Flox_R: 5'-GACCTGACAGAATAGGATATGCCCAACCTC-3'
5) For AMPKα2 conditional allele detection (Floxed band: 310bp, WT band: 260 bp)
AMPKA2_Flox_F: 5'-GCTTAGCACGTTACCCTGGAT-3'
AMPKA2_Flox_R: 5'-GTCTTCACTGAAATACATAGCA-3'
6) For sex detection (Y-specific SRY band: 402bp, unspecific band 544bp)[6]
SRY_F: 5'-TGGGACTGGTGACAATTGTC-3'
SRY_R: 5'-GAGTACAGGTGTGCAGCTCT-3'
IL3_F: 5'-GGGACTCCAAGCTTCAATCA-3'
IL3_R: 5'-TGGAGGAGGAAGAAAAGCAA-3'

DNA and plasmids

Endotoxin-free plasmid DNA was obtained using Macherey Nagel midi-prep kit. We used the following, previously described plasmids: empty vector pCAG-IRES-GFP (pCIG2), CRE-expressing vector pCAG-CRE-IRES-GFP (pCIG2-CRE)[7], mVENUS expressing vector pSCV2[8], pLKO, pCIG2-shSNPH, pCAG-mitoDsRed[9], pFUGW-PercevalHR[10], pCAG-mTagBFP2 and pCAG-mito-mTagBFP2 [11].

pCAG-mito-KillerRed was created by cloning the photosensitizer KillerRed targeted to the mitochondria with two mitochondria targeting sequences (MTS, derived from subunit 8 of human cytochrome C oxidase) into a pCAG vector between XhoI and NotI sites, following PCR amplification from a pCMV-mito-KillerRed plasmid (Evrogen). mScarlet-i cDNA was amplified by PCR from a pmScarlet-i_C1 plasmid (from Dorus Gadella, Addgene #85044)[12] and cloned into a pCAG2 backbone between XhoI and EcoRI sites. pCAG-mito-Grx1-roGFP2 and pCAG-cyto-Grx1-roGFP2[13], pCAG-CRE, and pCAG-vGlut1-GFP[14] were a kind gift from Tommy L Lewis (OMRF, USA), Seok-Kyu Kwon (KIST, Korea) and Etienne Herzog (U

Bordeaux, France), respectively. psPax2 and pMDG.2 (from Didier Trono, Addgene #12260 and #12259) were kindly provided by Patrick Lomonte (CNRS/PGNM, Lyon, France). pCAG-mito-eGFP was created by cloning the eGFP sequence from peGFP-C1 inside a pCAG-mito-DsRed[9] digested with XhoI and NotI to remove the DsRed sequence. Plasmids pCAG-mouseBRAWNIN-V5tag, pEF1alpha-Brawnin-IRES-GFP and pEF1alpha-IRES-GFP were created by VectorBuilder GmbH (Germany).

shRNAs

shRNAs against mouse NUAK1 were previously described[9]. shRNAs against mouse BRAWNIN (shRNA1: TRCN0000269184; shRNA2: TRCN0000269131) were selected from The RNAi Consortium shRNA Library (TRC) from the Broad Institute. The pLKO.1 TRC-cloning vector vas digested with AgeI-HF and EcoRI-HF, then purified with the NucleoSpin Gel and PCR clean up kit (Macherey-Nagel 740609.50). shRNA primers were annealed in NEBuffer 2 (NEB) and then ligated into the linearized pLKO.1 in rCutSmart buffer (NEB). The following oligonucleotide sequences were used:

ShRNA1 (TRCN0000269184)

F: 5'-CCGG-ATACCTGAAATTCCACCAAAG-CTCGAG-CTTTGGTGGAATTTCAGGTAT-TTTTTG-3'

R: 5'-AATTCAAAAA-ATACCTGAAATTCCACCAAAG-CTCGAG-CTTTGGTGGAATTTCAGGTAT-3'

ShRNA2 (TRCN0000269131)

F: 5'-CCGG-ACGCCACGAACCTCATGTTTC-CTCGAG-GAAACATGAGGTTCGTGGCGT-TTTTTG-3'

R: 5'-AATTCAAAAA-ACGCCACGAACCTCATGTTTC-CTCGAG- GAAACATGAGGTTCGTGGCGT-3'

Retro-transcription and PCR

RNA from Nuak1 KO cortical neurons and cortices was extracted using Trizol Reagent (Ambion by Life Technologies) and the PureLink RNA MiniKit 12183018a (Invitrogen). cDNA synthesis was done using 500ng of RNA with SuperScript II (Invitrogen) and Oligo(dT)12-18 (Invitrogen) primers.

Splicing PCR reaction (supplementary Figure 7) was done using Dream Taq DNA polymerase (ThermoScienfic) and C1000 Touch ThermoCycler (BioRad). The following primers were used:

mBRAWNIN_splice_F: 5'-TGTACTTTGGTTCCTGCCCG-3'

mBRAWNIN_splice_R: 5'-CGTGGCGTCTCTCTTTCAGT-3'

Following the splicing PCR, each band corresponding to an isoform of BRAWNIN was isolated using UV transilluminator (FLX-20M) and purified with NucleoSpin Gel and PCR

clean up (Macherey-Nagel 740609.50). Obtained DNA was then sequenced via Sanger method by EurofinGenomics.
qPCR reaction was performed using FastStart Universal SYBR Green Master Mix – ROX (Roche) and CFX Machine Connect Optics Module (BioRad).
The following primers were used:
mBRAWNIN_qPCR_F: 5'-CCAGGTGGTGCATTGGTACT-3'
mBRAWNIN_qPCR_R: 5'-GAAACATGAGGTTCGTGGCG-3'
RPL41_F: 5'-GCCATGAGAGCGAAGTGG-3'
RPL41_R: 5'-CTCCTGCAGGCGTCGTAG-3'
GUSB_F: 5'-GAGGATTGCCAACGAAACCG-3'
GUSB_R: 5'-GTGTCTGGGGACCACCTTTGA-3'
For gene expression analysis, delta-delta CT (threshold cycle) method was used[15]. The CT of the target gene was normalized to two control genes (RPL41 and GUSB).

Immunocytochemistry

Cells were fixed for 15 min at room temperature in 4% paraformaldehyde in PBS, washed 3 times in PBS, then permeabilized for 1 hr in Permeabilization buffer (PB: 0.3% Triton X-100, 0.3% BSA (Sigma), in PBS). Primary antibodies were incubated for 2 hr at room temperature in PB. Secondary antibodies were incubated for 1 hr at room temperature. Coverslips (BioCoat) were mounted on slides with Fluoromount G (EMS). Primary antibodies used for immunohistochemistry and immunocytochemistry in this study are polyclonal chicken anti-GFP (Rockland) (1:2000) and monoclonal rabbit anti-CRE (D7L7L) (1:1000, Cell Signaling). Secondary antibodies were polyclonal anti-chicken Alexa 488 (1:1000, Invitrogen) and polyclonal anti-rabbit Alexa 562 (1:1000, Invitrogen). Nuclear DNA was stained using Hoechst 33258 (1:10000, Pierce).

Ratiometric probes signal quantification

For experiments using the biosensor PercevalHR, we used sequentially a blue (405nm - $Perceval_{blue}$) and green (488nm - $Perceval_{green}$) laser, and acquired images in 1024x1024 mode using the Nikon C2 confocal microscope. We acquired one frame every minute for 15 minutes to measure the basal signal (F_0), then added FCCP (2µM) and imaged for 25 more minutes to determine the minimal signal (F_{min}). ROIs were drawn in the soma, axon and outside of the cell (for background correction). Signal was defined as the ratio: PercevalHR = ($Perceval_{green}$ – background) / ($Perceval_{blue}$ – background). We next expressed the basal

signal as a fraction of the minimal signal by the following conversion F_0/F_{min}, which is presented in Figure 3 J and K. Raw values of F_0 and F_{min} are presented in Supplementary Figure 11.

For experiments using roGFP2, we used sequentially a blue (405nm - $roGFP2_{blue}$) and green (488nm - $roGFP2_{green}$) laser, and acquired images in 1024x1024 mode using the Nikon C2 confocal microscope. Signal was defined as the ratio: roGFP2 = ($roGFP2_{blue}$ – background) / ($roGFP2_{green}$ – background). We normalized the signal to the average of WT condition to account for inter-culture variability.

For TMRE measurements, we used a 561nm laser and acquired images in 1024x1024 mode using the Nikon C2 confocal microscope. We acquired one frame every 30 seconds for 5 minutes to measure the basal signal (F_0), then added FCCP (2μM) and imaged for 10 more minutes to determine the minimal signal (F_{min}). ROIs were drawn around axonal mitochondria and outside of the cell (for background correction). Signal was defined as following: $F_{TMRE} = F_0 - F_{min}$. We normalized the signal to the average of WT condition to account for inter-culture variability.

Western blotting

Total proteins from HeLa cells were extracted with a lysis buffer containing 50 mM Tris, pH 8; 550 mM NaCl; 2% NP40; 0,1% SDS and 0,5% sodium deoxycholate (For total AMPK). Brain cortical samples were mechanically-dissociated with plastic pestles in the following lysis buffer: 50 mM Tris, pH 7,5; 600mM NaCl; 1% NP-40; 0,1% SDS; 0,5% sodium deoxycholate. Protease inhibitor cocktail (SIGMA*FAST*; Sigma-Aldrich) and phosphatase inhibitor cocktail (PhosSTOP; Roche) were added according to manufacturer’s instructions. Cell lysates were sonicated, incubated for 30 min on ice and then centrifuged at 20,000 g for 5 min to remove cell debris. Protein concentration was determined with a colorimetric Lowry-like assay (DC protein assay kit, Bio-Rad). Equal amounts of proteins (10-20 μg) were then separated by SDS-PAGE and transferred onto PVDF Immobilon-P membranes (Millipore). Membranes were blocked with TBST (Tris-buffered saline [50 mM Tris, 150 mM NaCl, pH 7.4] + 0.1% Tween 20) containing 5% bovine serum albumin (Euromedex) and incubated overnight at 4°C with primary antibodies. Membranes were washed 3 times in TBST for 10 min and incubated for 1h with horseradish peroxidase-conjugated secondary antibodies. After 3 washes in TBST, membranes were incubated with enhanced chemiluminescence reagents (Amersham ECL, GE Healthcare) for 5 minutes and signals were acquired using Chemidoc technology. Quantification of band intensities was made with ImageJ software.

Antibodies for Western blotting

The following commercial primary antibodies were used in this study: mouse anti-Actin antibody (C4 clone) (1:5000, MP Biomedicals), rabbit anti phospho-AMPKα (Thr172) (D4D6D) (1:800, Cell Signaling Technology), rabbit anti-AMPKα (D63G4) (1:1000, Cell Signaling Technology), sheep anti-phospho-NUAK1 Thr211 (S234B) with non phosphopeptide NUAK1 (Thr211) (1:1000, MRC Protein Phosphorylation Unit, United Kingdom), rabbit anti-NUAK1 (E4T2A) (1:1000, Cell Signaling Technology), rabbit anti-LKB1 (D60C5) (Cell Signaling Technology), rabbit anti-OPA1 (1:2000, Abcam), mouse anti-MFN1 (11E91H12) (1:1000, Abcam), mouse anti-MFN2 (6A8) (1:1000, Abcam), mouse anti-DRP1 (Clone 8/DLP1) (1:2000, BD Biosciences), mouse anti-PGC1α (4C1.3) (1:1500, Millipore), mouse anti-beta-Tubulin 3/Tuj1 (GT11710) (1:5000, GeneTex), rabiit anti-TOM20 (D8T4N) (1:5000, Cell Signaling Technology), polyclonal anti-LC3 (1:1000, Cell Signaling Technology 2775) and mouse anti-V5 (SV5-Pk1) (1:5000, Invitrogen). Horseradish peroxidase-coupled secondary polyclonal antibodies were goat anti-mouse IgG and Sheep Ig (Invitrogen) and donkey anti-rabbit (Novex, Lifetechnologies).

Metabolomic flux analyzes

We used a Seahorse XFe24 (NUAK1 constitutive KO neurons – Figure 3) and XFe96 analyzers (all other experiments – Figure 8, Supplementary figure 3, Supplementary figure 7) (Agilent) for oxygen consumption and extracellular acidification measurements with the Glycolysis and Mito Stress test kits. Neurons were plated at 100,000 cells per well in Seahorse compatible 24-well plates or at 70 000 cells per well in Seahorse compatible 96-well plates. Metabolic activity was measured at 7 days in vitro (24-well plates assay) or 5 days in vitro (96-well plates assay). The assay was conducted in Modified HBSS (1XHBSS supplemented with glucose (3.5g/L), Sodium pyruvate 1mM, Glutamax 1X, CaCl2 1mM, MgSO4 1mM, pH 7.4). Following measurement, for 24-well plates, neurons were lysed in ice-cold lysis buffer (150mM NaCl, 0,5M EDTA ph8, 50mM Tris pH 8; 1% NP-40, 0.5% sodium deoxycolate, 0.1% SDS) and protein concentration was measured by Bradford for post-hoc normalization. For 96-well plates, neurons were fixed for 10 minutes in PFA 4% containing TritonX100 (0.3%) and Hoechst 33258 (1:10 000, Pierce) and imaged with an EVOS M5000. Nuclei number was counted per microchamber per well using FIJI Stardist plugin[16] and used for OCR normalization.

Mitochondria DNA

Mitochondrial DNA was measured by qPCR following a protocol described elsewhere (Oruganty-Das et al., 2012). 7DIV primary neurons DNA was extracted by whole cell lysis in lysis buffer (Tris pH8 100mM, EDTA 5mM, SDS 0.2%, NaCl 200mM, proteinase K 0.5mg/mL) followed by isopropanol precipitation. Quantitative PCR was performed with the CFX-connect thermal cycler (BioRad). The following primers were used: mtDNA_F: ACCATTTGCAGACGCCATAA, mtDNA_R: TGAAATTGTTTGGGCTACGG, betatub_F: GCCAGAGTGGTGCAGGAAA, betatub_R: TCACCACGTCCAGGACAG.

Biochemical Assay

Biochemical assays were performed on mouse cortex samples from NUAK1 KO or NUAK1 WT mice. Samples were subjected to a chemical lysis using RIPA buffer (150mM NaCl, 0,5M EDTA ph8, 50mM Tris pH 8; 1% NP-40, 0.5% sodium deoxycolate, 0.1% SDS) and followed by a mechanical lysis using the Precellys Evolution.

Citrate Synthase enzymatic activity (EA) was determined by measuring absorbance using a PowerWave XS plate reader (BioTek). We used the Beer Lambert law EA= Mean V(ε x l) where l is the width of the tank (l = 0.4 cm), ε = 13600 uA/M/cm and Mean V was expressed in mDO/min. CS activity was measured from a volume of 20µL (100µg of total proteins) diluted in 90µL of specific reaction buffer (65µL of 50mM KH2PO4, pH 7.5, 10µL of Acetyl-CoA 4.4mM, 10µL of Oxaloacetate 4.4mM, 10µL of 150mM DTNB). Absorbance at 600nm was performed over 15 minutes at room temperature (one read per 10 seconds).

Lentiviral production and infection

HEK293T cells were plated in 60-mm dishes at 1.2^10^6 cells per plate the day before the transfection. Cell transfections were performed with psPax, pMDG.2 and pLKO.1/pLKO-shBRAWNIN using the calcium phosphate method. Medium was replaced 12 hours post-transfection. Supernatants were collected 48 hours post-transfection and filtered through low-protein-binding 0.45-µm filters (Millipore), precipitated with PEG-It 5X (System Biosciences) overnight at 4 degrees and centrifuged for 30 minutes at 4 degrees 1500g. Neurons were transduced with the multiplicity of infection of 5 at 1DIV. The medium was replaced once at 2DIV.

L-Carnitine supplementation to animals

For the L-Carnitine supplementation experiment, pregnant mice were treated with veterinary-approved L-Carnitine (Isulik 20, Sogeval laboratories) in the drinking water since detection of

gestation (E13.5). Drug treatment was continued after birth and during lactation, and pups were sacrificed at weaning (P21) when cortical layer 2/3 PNs axons display complete branching patterns.

RNA sequencing

RNA-Seq libraries for poly-A-enriched fractions were generated from total RNA using TruSeq Stranded mRNA Library Prep Kit and TruSeq RNA Single Indexes kits A and B (Illumina, San Diego, CA), according to manufacturer's instructions. High throughput sequencing (1x50 and 2x100 base reads for in vitro and in vivo experiments, respectively) was carried out on an Illumina HiSeq 4000 platform (GenomEast Platform, a member of the "France Génomique" consortium - ANR-10-INBS-0009, Illkirch, France).

In vivo RNA-seq: Cutadapt (1.13)[17] and Prinseq (0.20.4) were used for read preprocessing: adapter and low-quality (Phred quality score below 20) bases trimming, removal of reads shorter than 25bp after trimming. Reads were then mapped into the mm9 assembly of the mouse genome using Tophat2 (2.0.13)[18] and Samtools (0.1.19)[19]. Gene expression was quantified using HTSeq-count[20] and differential gene expression analysis was carried out using the DESeq2 package[21], with the following parameters: $|Log2FC| > 0.25$, $p < 0.05$.

In vitro RNA-seq: Cutadapt[17] version 1.10 was used for read preprocessing: adapter and low-quality (Phred quality score below 20) bases trimming, removal of reads shorter than 40 bp after trimming. Reads mapping to rRNA were also discarded. Reads were then mapped onto the mm10 assembly of the mouse genome using STAR[22] version 2.5.3a. Gene expression was quantified from uniquely aligned reads using HTSeq-count[20] version 0.6.1p1 with gene annotations from Ensembl release 92 and union mode. Statistical analysis was performed using R 3.3.2 and DESeq2 1.16.1 Bioconductor library[21]. More precisely, counts were normalized from the estimated size factors using the median ratio method and a Wald test was used for the statistical test. Unwanted variation was identified using SVA[23] and considered in the statistical model. To reduce false positive, p-values were adjusted by IHW method[24].

Transcriptional analysis

Both “over representation analysis” (ORA) and “gene set enrichment analysis” (GSEA) were done using the R package “Cluster Profiler” (https://guangchuangyu.github.io/2016/01/go-analysis-using-clusterprofiler/)[25].

ORA analysis of in vivo and in vitro datasets was based on identification of DEG, extracted by using the following criteria: Log2FC.threshold > 0.25, pvalue < 0,05, to interrogate the following databases: Gene Ontology (Biological Processes) "c5.go.bp.v7.5.1.symbols.gmt", Kegg "c2.cp.kegg.v7.5.1.symbols.gmt", and Reactome "c2.cp.reactome.v7.5.1.symbols.gmt". GSEA analysis was performed on the ranked list of all protein coding genes, using the GSEA software v4.1.0 [Built 27] from the Broad institute (GSEA (gsea-msigdb.org)). In addition to the databases used for the ORA analysis, the following databases were included for this analysis: Hallmarks "hall.v7.5.1.symbols.gmt", and Wikipathway "c2.cp.wikipathways.v7.5.1.symbols.gmt". Significantly affected gene sets were sorted using the following criteria: FDR qvalue < 0,05. Obtained results were exported in cytoscape 3.9.1 for visualization and analysis. (www.cytoscape.org). For comparison of in vivo and in vitro results, significant gene sets were manually assigned to thematic categories.

SUPPLEMENTARY REFERENCES

1. Courchet, V. *et al.* Haploinsufficiency of autism spectrum disorder candidate gene NUAK1 impairs cortical development and behavior in mice. *Nature communications* **9**, 4289 (2018).

2. Goebbels, S. *et al.* Genetic targeting of principal neurons in neocortex and hippocampus of NEX-Cre mice. *Genesis (New York, N.Y. : 2000)* **44**, 611–621 (2006).

3. Viollet, B. *et al.* The AMP-activated protein kinase α2 catalytic subunit controls whole-body insulin sensitivity. *J Clin Invest* **111**, 91–98 (2003).

4. Jørgensen, S. B. *et al.* Knockout of the α2 but Not α1 5′ -AMP-activated Protein Kinase Isoform Abolishes 5-Aminoimidazole-4-carboxamide-1-β-4-ribofuranosidebut Not Contraction-induced Glucose Uptake in Skeletal Muscle*. *J Biol Chem* **279**, 1070–1079 (2004).

5. Boudaba, N. *et al.* AMPK Re-Activation Suppresses Hepatic Steatosis but its Downregulation Does Not Promote Fatty Liver Development. *Ebiomedicine* **28**, 194–209 (2018).

6. Lambert, J.-F. *et al.* Quick sex determination of mouse fetuses. *J. Neurosci. Methods* **95**, 127–132 (2000).

7. Hand, R. *et al.* Phosphorylation of Neurogenin2 specifies the migration properties and the dendritic morphology of pyramidal neurons in the neocortex. *Neuron* **48**, 45–62 (2005).

8. Hand, R. & Polleux, F. Neurogenin2 regulates the initial axon guidance of cortical pyramidal neurons projecting medially to the corpus callosum. *Neural development* **6**, 30 (2011).

9. Courchet, J. *et al.* Terminal axon branching is regulated by the LKB1-NUAK1 kinase pathway via presynaptic mitochondrial capture. *Cell* **153**, 1510–1525 (2013).

10. Tantama, M., Martínez-François, J. R., Mongeon, R. & Yellen, G. Imaging energy status in live cells with a fluorescent biosensor of the intracellular ATP-to-ADP ratio. *Nature communications* **4**, 2550 (2013).

11. Lewis, T. L., Turi, G. F., Kwon, S.-K., Losonczy, A. & Polleux, F. Progressive Decrease of Mitochondrial Motility during Maturation of Cortical Axons In Vitro and In Vivo. *Current biology : CB* **26**, 2602–2608 (2016).

12. Bindels, D. S. *et al.* mScarlet: a bright monomeric red fluorescent protein for cellular imaging. *Nature methods* **14**, 53–56 (2017).

13. Gutscher, M. *et al.* Real-time imaging of the intracellular glutathione redox potential. *Nature methods* **5**, 553–559 (2008).

14. Herzog, E. *et al.* In vivo imaging of intersynaptic vesicle exchange using VGLUT1 Venus knock-in mice. *The Journal of neuroscience : the official journal of the Society for Neuroscience* **31**, 15544–15559 (2011).

15. Livak, K. J. & Schmittgen, T. D. Analysis of Relative Gene Expression Data Using Real-Time Quantitative PCR and the 2−ΔΔC T Method. *Methods* **25**, 402–408 (2001).

16. Schmidt, U., Weigert, M., Broaddus, C. & Myers, G. Medical Image Computing and Computer Assisted Intervention – MICCAI 2018, 21st International Conference, Granada, Spain, September 16-20, 2018, Proceedings, Part II. *Lect. Notes Comput. Sci.* 265–273 (2018) doi:10.1007/978-3-030-00934-2_30.

17. Martin, M. Cutadapt removes adapter sequences from high-throughput sequencing reads. *Embnet J* **17**, 10–12 (2011).

18. Kim, D. *et al.* TopHat2: accurate alignment of transcriptomes in the presence of insertions, deletions and gene fusions. *Genome Biol* **14**, R36 (2013).

19. Danecek, P. *et al.* Twelve years of SAMtools and BCFtools. *Gigascience* **10**, giab008 (2021).

20. Anders, S., Pyl, P. T. & Huber, W. HTSeq—a Python framework to work with high-throughput sequencing data. *Bioinformatics* **31**, 166–169 (2015).

21. Love, M. I., Huber, W. & Anders, S. Moderated estimation of fold change and dispersion for RNA-seq data with DESeq2. *Genome Biol* **15**, 550 (2014).

22. Dobin, A. *et al.* STAR: ultrafast universal RNA-seq aligner. *Bioinformatics* **29**, 15–21 (2013).

23. Leek, J. T. svaseq: removing batch effects and other unwanted noise from sequencing data. *Nucleic Acids Res* **42**, e161–e161 (2014).

24. Ignatiadis, N., Klaus, B., Zaugg, J. B. & Huber, W. Data-driven hypothesis weighting increases detection power in genome-scale multiple testing. *Nat Methods* **13**, 577–580 (2016).

25. Yu, G., Wang, L.-G., Han, Y. & He, Q.-Y. clusterProfiler: an R package for comparing biological themes among gene clusters. *Omics : a journal of integrative biology* **16**, 284–287 (2012).
