# Supplementary material for: The AMPK-related kinase NUAK1 controls cortical axons branching by locally modulating mitochondrial metabolic functions
Source: Nat Commun. 2024 Mar 21;15:2487. doi: 10.1038/s41467-024-46146-6 (PMC10958033; doi:10.1038/s41467-024-46146-6)
Supplement: Supplementary file 3 — Description of Additional Supplementary Files [file 41467_2024_46146_MOESM3_ESM.pdf]

File name: Supplementary Data 1
Description: Full list of DEGs for in vivo and in vitro transcriptional analysis of NUAK1 KO vs WT mice cortex and cultured neurons. DEGs are presented with related p values. Related to Figure 6 and S8.

File name: Supplementary Data 2
Description: Gene ontology (Biological processes), Kegg and Reactome over-representation analysis for transcripts enriched in NUAK1 KO vs WT mice cortex (in vivo) and cultured neurons (in vitro). Categories selected for bar graphs in Fig6C and Fig S8B are highlighted. Related to Figure 6 and S8.

File name: Supplementary Data 3
Description: Curated gene set enrichment analysis of NUAK1 KO vs WT mice cortex (in vivo) and cultured neurons (in vitro). Genesets were hand-curated into thematic categories organized in separated sheets. Related to Figure 7.

File name: Supplementary Movie 1
Description: (related to Figure 1): recruitment of mitochondria at the origin and inside a stable branch. Axon (green) was visualized by mVenus expression. Mitochondria were marked with mito-DsRed. Real movie time: 3 hours 55 minutes.
