# Supplementary material for: The AMPK-related kinase NUAK1 controls cortical axons branching by locally modulating mitochondrial metabolic functions
Source: Nat Commun. 2024 Mar 21;15:2487. doi: 10.1038/s41467-024-46146-6 (PMC10958033; doi:10.1038/s41467-024-46146-6)
Supplement: Supplementary file 8 — Reporting Summary [file 41467_2024_46146_MOESM8_ESM.pdf]

nature portfolio

Corresponding author(s): Julien COURCHET
Manuscript NCOMMS-23-13021

Last updated by author(s): Jan 31, 2024

Reporting Summary

Nature Portfolio wishes to improve the reproducibility of the work that we publish. This form provides structure for consistency and transparency in reporting. For further information on Nature Portfolio policies, see our Editorial Policies and the Editorial Policy Checklist.

Statistics

For all statistical analyses, confirm that the following items are present in the figure legend, table legend, main text, or Methods section.

n/a	Confirmed	
☐	☒	The exact sample size (*n*) for each experimental group/condition, given as a discrete number and unit of measurement
☐	☒	A statement on whether measurements were taken from distinct samples or whether the same sample was measured repeatedly
☐	☒	The statistical test(s) used AND whether they are one- or two-sided<br>*Only common tests should be described solely by name; describe more complex techniques in the Methods section.*
☐	☒	A description of all covariates tested
☐	☒	A description of any assumptions or corrections, such as tests of normality and adjustment for multiple comparisons
☒	☐	A full description of the statistical parameters including central tendency (e.g. means) or other basic estimates (e.g. regression coefficient) AND variation (e.g. standard deviation) or associated estimates of uncertainty (e.g. confidence intervals)
☐	☐	For null hypothesis testing, the test statistic (e.g. *F*, *t*, *r*) with confidence intervals, effect sizes, degrees of freedom and *P* value noted<br>*Give P values as exact values whenever suitable.*
☒	☐	For Bayesian analysis, information on the choice of priors and Markov chain Monte Carlo settings
☒	☐	For hierarchical and complex designs, identification of the appropriate level for tests and full reporting of outcomes
☒	☐	Estimates of effect sizes (e.g. Cohen's *d*, Pearson's *r*), indicating how they were calculated

Our web collection on statistics for biologists contains articles on many of the points above.

Software and code

Policy information about availability of computer code

Data collection	Imaging data was collected using the NIS Elements software (version 4.6)
Data analysis	Statistical analyses were performed using Prism version 9.5 (GraphPad)<br>Imaging data was analyzed using the NIS Elements software (version 4.6)<br>Softwares used for RNAseq analysis and gene-expression analyses are described in full in the supplementary methods section of the paper, including reference to the original papers and when applicable link to the online tools.

For manuscripts utilizing custom algorithms or software that are central to the research but not yet described in published literature, software must be made available to editors and reviewers. We strongly encourage code deposition in a community repository (e.g. GitHub). See the Nature Portfolio guidelines for submitting code & software for further information.

Data

Policy information about availability of data

All manuscripts must include a data availability statement. This statement should provide the following information, where applicable:

- Accession codes, unique identifiers, or web links for publicly available datasets
- A description of any restrictions on data availability
- For clinical datasets or third party data, please ensure that the statement adheres to our policy

Data used to generate all quantification results presented in the figures of this manuscript are available upon reasonable request to the corresponding author.
Source data are provided with this paper as a Source Data file.
Transcriptomic data presented in Figure 6, 7 and Supplementary Figure 8 has been deposited on GEO.
- RNAseq data ("in vitro") can be accessed on GEO with accession code GSE227203 [https://www.ncbi.nlm.nih.gov/geo/query/acc.cgi?acc=GSE227203]
- RNAseq data ("in vivo") can be accessed on GEO with accession code GSE226698 [https://www.ncbi.nlm.nih.gov/geo/query/acc.cgi?acc=GSE226698]

Research involving human participants, their data, or biological material

Policy information about studies with human participants or human data. See also policy information about sex, gender (identity/presentation), and sexual orientation and race, ethnicity and racism.

Reporting on sex and gender	No human participant or biological material from human participants origin was used in this study
Reporting on race, ethnicity, or other socially relevant groupings	No human participant or biological material from human participants origin was used in this study
Population characteristics	No human participant or biological material from human participants origin was used in this study
Recruitment	No human participant or biological material from human participants origin was used in this study
Ethics oversight	No human participant or biological material from human participants origin was used in this study

Note that full information on the approval of the study protocol must also be provided in the manuscript.

Field-specific reporting

Please select the one below that is the best fit for your research. If you are not sure, read the appropriate sections before making your selection.

☒ Life sciences ☐ Behavioural & social sciences ☐ Ecological, evolutionary & environmental sciences

For a reference copy of the document with all sections, see nature.com/documents/nr-reporting-summary-flat.pdf

Life sciences study design

All studies must disclose on these points even when the disclosure is negative.

Sample size	Sample size was determined empirically based on our previous experience and standards from the field.
Data exclusions	Unless specified in the methods or legend section, no data was excluded after quantification.
Replication	Number of independent replicates is indicated in the legend for each figures. For primary neuronal cultures (in vitro), we considered that independent experiments are cultures performed on different days, and/or coming from distinct animals. For in utero cortical electroporations (in vivo), each animal was considered independent, although we systematically include in the experiment animals coming from distinct litters/distinct mothers.
Randomization	Not applicable
Blinding	Whenever possible, quantifications were performed blind to the genotype.

Reporting for specific materials, systems and methods

We require information from authors about some types of materials, experimental systems and methods used in many studies. Here, indicate whether each material, system or method listed is relevant to your study. If you are not sure if a list item applies to your research, read the appropriate section before selecting a response.

Materials & experimental systems

n/a	Involved in the study
☐	☒ Antibodies
☐	☒ Eukaryotic cell lines
☒	☐ Palaeontology and archaeology
☐	☒ Animals and other organisms
☒	☐ Clinical data
☒	☐ Dual use research of concern
☒	☐ Plants

Methods

n/a	Involved in the study
☒	☐ ChIP-seq
☒	☐ Flow cytometry
☒	☐ MRI-based neuroimaging

Antibodies

Antibodies used

For all antibodies, information regarding the specie, dilution, clone number and provider are indicated in the Methods section.
The following commercial primary antibodies were used in this study:
mouse anti-Actin antibody (C4 clone) (1:5000, MP Biomedicals),
rabbit anti phospho-AMPKα (Thr172) (D4D6D) (1:800, Cell Signaling Technology),
rabbit anti-AMPKα (D63G4) (1:1000, Cell Signaling Technology),
sheep anti-phospho-NUAK1 Thr211 (S234B) with non phosphopeptide NUAK1 (Thr211) (1:1000, MRC Protein Phosphorylation Unit, United Kingdom),
rabbit anti-NUAK1 (E4T2A) (1:1000, Cell Signaling Technology),
rabbit anti-LKB1 (D60C5) (Cell Signaling Technology),
rabbit anti-OPA1 (1:2000, Abcam),
mouse anti-MFN1 (11E91H12) (1:1000, Abcam),
mouse anti-MFN2 (6A8) (1:1000, Abcam),
mouse anti-DRP1 (Clone 8/DLP1) (1:2000, BD Biosciences),
mouse anti-PGC1α (4C1.3) (1:1500, Millipore),
mouse anti-beta-Tubulin 3/Tuj1 (GT11710) (1:5000, GeneTex),
rabbit anti-TOM20 (D8T4N) (1:5000, Cell Signaling Technology),
polyclonal anti-LC3 (1:1000, Cell Signaling Technology 2775)
mouse anti-V5 (SV5-Pk1) (1:5000, Invitrogen).
Secondary antibodies:
Hoechst 33258 solution (Merk/Sigma Aldrich)
Goat Anti-mouse HRP (Invitrogen)
Donkey Anti-Rabbit HRP (Invitrogen)
Goat Anti-Chicken AlexaFluor Plus 488 (Invitrogen)
Donkey Anti-Mouse AlexaFluor 647 (Invitrogen)

Validation

For commercial antibodies, validation was according to the manufacturer's datasheet. We systematically verify the specificity of antibodies upon purchase by western-blot and/or immunofluorescence against appropriate positive and negative control samples (samples expressing the protein or not).

Eukaryotic cell lines

Policy information about cell lines and Sex and Gender in Research

Cell line source(s)

All cell lines used in this study were purchased from ATCC

Authentication

None of the cell lines were authenticated as we relied on the ATCC's stock

Mycoplasma contamination

All cell lines were tested for mycoplasma contamination on a regular basis and all tests were negative

Commonly misidentified lines (See ICLAC register)

Name any commonly misidentified cell lines used in the study and provide a rationale for their use.

Animals and other research organisms

Policy information about studies involving animals; ARRIVE guidelines recommended for reporting animal research, and Sex and Gender in Research

Laboratory animals

Description of the animals can be found in the methods section of the manuscript.

Wild animals

No wild animals were used and included in this study

Reporting on sex

From our experience, sex is not a factor influencing the phenotype in young animals and developing neurons in vitro. Hence, we did not collect data to discriminate sex of the animals for culture. For in vivo experiments (ie. in utero cortical electroporations), males and females were included regardless of sex. No apparent sex effect could be detected.

Field-collected samples	No field collected samples were used and included in this study
Ethics oversight	Mice breeding and handling was performed by following National Institutes of Health guidelines and the French and European legislation. Experimental protocols were approved by the CECCAPP Ethics committee (C2EA15) of the University of Lyon.

Note that full information on the approval of the study protocol must also be provided in the manuscript.
